# The Value of Anterior Segment Optical Coherence Tomography in Different Types of Corneal Infections: An Update

**DOI:** 10.3390/jcm10132841

**Published:** 2021-06-27

**Authors:** Ahmed A. Abdelghany, Francesco D’Oria, Jorge Alio Del Barrio, Jorge L. Alio

**Affiliations:** 1 Ophthalmology Department, Faculty of Medicine, Minia University, Minia 61519, Egypt; ahmeda.abdelghany82@gmail.com; 2 Section of Ophthalmology, Department of Basic Medical Sciences, Neurosciences and Sense Organs, University of Bari, 70124 Bari, Italy; francescodoria91@hotmail.it; 3 Vissum Miranza, Miguel Hernandez University, c/Cabañal, 1, 03016 Alicante, Spain; jorge_alio@hotmail.com

**Keywords:** corneal infection, anterior segment, optical coherence tomography

## Abstract

Anterior segment optical coherence tomography (AS-OCT) is a modality that uses low-coherence interferometry to visualize and assess anterior segment ocular features, offering several advantages of being a sterile and noncontact modality that generates high-resolution cross-sectional images of the tissues. The qualitative and quantitative information provided by AS-OCT may be extremely useful for the clinician in the assessment of a wide spectrum of corneal infections, guiding in the management and follow-up of these patients. In clinical practice, infections are routinely evaluated with slit-lamp biomicroscopy, an examination and imaging modality that is limited by the physical characteristics of light. As a consequence, the depth of pathology and the eventually associated corneal edema cannot be accurately measured with the slit-lamp. Therefore, it represents a limit for the clinician, as in vivo information about corneal diseases and the response to treatment is limited. Resolution of corneal infection is characterized by an early reduction in corneal edema, followed by a later reduction in infiltration: both parameters can be routinely measured with standardized serial images by AS-OCT.

## 1. Introduction

**Figure 1:**
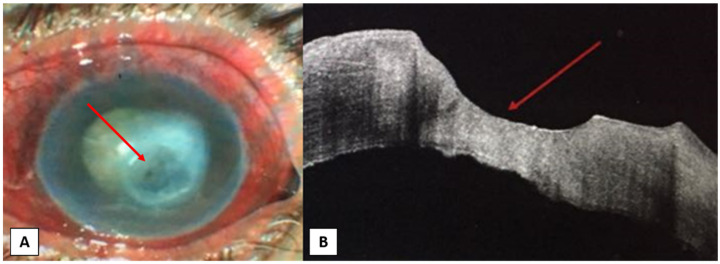
A female patient, 60-year-old, with an infected neurotrophic corneal ulcer. (**A**) Slit-lamp photo showing paracentral corneal ulcer (red arrow) with an area of corneal opacity above it and total lens opacity. (**B**) AS-OCT photo showing the absence of epithelium over ulcer area (red arrow), thin hyperreflective corneal stroma in the base of ulcer area with areas of irregular endothelium.

Keratitis is an inflammatory condition of the cornea that determines a change in its structure and a reduction in its transparency [1]. Infectious keratitis is one of the major causes of preventable blindness in developing and developed countries [1,2]. Its main signs are corneal epithelial ulceration, corneal edema, and stromal infiltration with the presence of inflammatory cells (Figure 1) [3,4,5,6].

**Figure 2:**
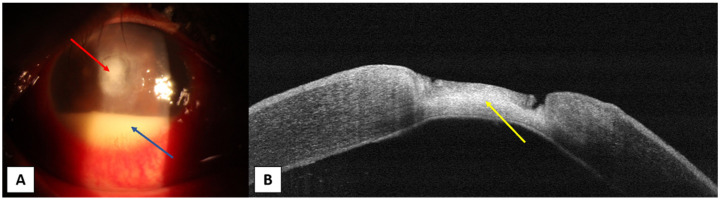
A male patient, 50-year-old, with history of trauma in the right eye. (**A**) Slit-lamp photo showing central rounded corneal ulcer (red arrow) with about 4 mm hypopyon in the anterior chamber (blue arrow). (**B**) OCT photo showing absent epithelium over ulcer area, ulcer base is irregular and stroma under the ulcer base is hyperreflective (yellow arrow).

Slit-lamp biomicroscopy is the routine tool clinically used for the examination of keratitis to assess the site of infiltrates and measure the horizontal and vertical dimensions of the epithelial ulceration and infiltration (Figure 2).

This examination modality has disadvantages as it is limited by the physical characteristics of light and has a difficulty in measuring the depth of the corneal infiltration and associated corneal edema [7]. As a result, in vivo information about corneal changes and tissue response to the treatment is limited. Subjective assessment such as the improvement of symptoms could point out that the infection is being resolved.

Nevertheless, keratitis can be assessed in vivo with anterior segment optical coherence tomography (AS-OCT). This imaging modality provides cross-sectional scans of the cornea, helping to evaluate the depth of inflammation through measurements of stromal infiltration thickness (IT) and corneal thickness (CT) [8]. AS-OCT has revolutionized the clinical and surgical management of the cornea, giving a new perspective on corneal pathology.

This literature review aims to show the value of AS-OCT in vivo assessment and monitoring of various types of corneal infections, reviewing the available evidence on this subject in the application of AS-OCT in infectious keratitis.

## 2. Corneal Infections

Infective keratitis can be caused by different microorganisms such as bacteria, fungi, and protozoa (Acanthamoeba) [6]. The abundance of microorganisms in different geographical locations differs because of differences in demographic factors, predisposing risk factors, socioeconomic conditions, and climate [9]. History of trauma, ocular surface disorders, corneal exposure, and contact lens wear are possible risk factors. There are also iatrogenic risk factors such as any ocular surgery and topical steroids [10]. 

**Figure 3:**
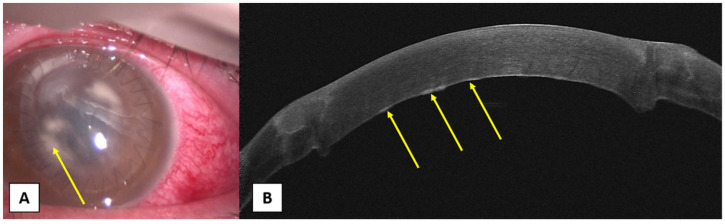
(**A**) Slit-lamp photo showing multiple whitish infiltrates with less defined margins (yellow arrow). (**B**) AS-OCT image showing the early healing pattern of a mushroom-configuration DALK with multiple areas of hyperreflectivity at the graft–host interface (yellow arrows). Absence of anterior chamber involvement and the cornea is swollen. Therapeutic penetrating keratoplasty was carried out in addition with cultures of the donor lenticule removal and the patient was treated with systemic Tigecycline and Linezolid according to the antibiogram.

Bacteria are the prevalent cause of infective keratitis in temperate countries [9,11,12]. Pseudomonas species are responsible for most of contact lens-related bacterial keratitis; other common Gram-negative bacteria include *Serratia marcescens* and Enterobacteriaceae (Figure 3).

If left untreated, corneal necrosis, thinning and perforation may occur [6]. Gram-positive cocci, many of which are commensals of the skin as well as conjunctiva, may cause infective keratitis. That is the case of Staphylococcus and Streptococcus species. Other less common causes of bacterial keratitis include atypical Mycobacterium and Nocardia species [6]. Fungus is a crucial cause of infective keratitis in tropical countries often due to trauma by vegetable matter [6,9]. In developed countries, it occurs mostly in eyes with ocular surface disease, immunosuppression, or contact lens wear [6]. It can be caused by filamentary fungi (e.g., Fusarium, Aspergillus) or yeasts (e.g., Candida). Microsporidia is another fungal organism that is emerging as a cause of fungal keratitis [13,14]. Finally, acanthamoeba keratitis is mostly related to soft contact lens use and the abundance of water contamination and swimming pools by acanthamoeba [15]. 

Patients with infective keratitis are usually symptomized by an acute onset of pain, photophobia, red eye, and varying degrees of visual loss depending on the severity and location of the corneal lesion. A detailed history can identify risk factors and possible etiology. Slit lamp examination is still the most important diagnostic tool [16]: bacterial keratitis mostly shows a well-defined corneal infiltrate. Soft or serrated infiltrates and satellite lesions may suggest a fungal cause. Acanthamoeba keratitis is characterized by the presence of perineural infiltrates, dendritiform lesions, and later ring infiltrates [6]. In addition to biomicroscopic examination, corneal scrapes taken from the ulcer edge are essential to identify the pathogenic organism. These samples are sent for microscopy (Gram stain), culture, and sensitivity testing [17]. PCR is also used to identify the pathogenic organism and is more sensitive than regular culture techniques [18].

## 3. Anterior Segment Optical Coherence Tomography 

OCT is a non-contact in vivo ocular imaging technology using low coherence interferometry that was initially developed for retinal imaging by Huang et al. in 1991 [19]. OCT measures the echo time delay of light backscattered from tissue structures, hence it produces two- and three-dimensional cross-sectional imaging of tissue [20]. OCT was traditionally used for visualization of the posterior segment until the first application for AS-OCT in 1994. It is faster, less invasive, and more patient-friendly than other anterior segment imaging devices such as ultrasonic biomicroscopy [21]. Time-domain and frequency-domain OCT are the two broad categories of AS-OCT. Frequency-domain OCT can be further divided into Fourier-domain OCT (spectral-domain OCT) and swept-source OCT (SS-OCT) [19]. 

Time-domain OCT. The two most common available devices of time-domain OCT (TD-OCT) are the Visante OCT (Carl Zeiss Meditec, Oberkochen, Germany), and Heidelberg slit lamp OCT (SL-OCT, Heidelberg Engineering GmbH, Heidelberg, Germany) [22]. Visante OCT has a wavelength of 1310 nm, a 16 mm scan width, and 6 mm scan depth. Axial resolution 18 μm and transverse resolution is 60 μm [22]. Heidelberg SL-OCT also has a 1310 nm wavelength as well as a 15 mm width, and 7 mm scan depth. Axial resolution is >25 μm and transverse resolution is 20–100 μm [23]. Major drawbacks of TD-OCT relate to the low A-scan rate (2000 A-scans/second with the Visante OCT system, and 200 A-scans/second with the SL-OCT) [21].

Spectral-domain OCT. Spectral-domain OCT (SD-OCT) generates varying wavelengths of light, with a higher speed of acquisition (10 to 100 times that of TD-OCT) [24]. SD-OCT machines include the Spectralis (Heidelberg Engineering GmbH), RTVue (Optovue, Inc., Fremont, CA, USA) and Cirrus OCT (Carl Zeiss Meditec) [25]. Other variations of SD-OCT are custom-built designs that have the advantage of improved axial resolution, termed as “ultrahigh-resolution OCT”. Reported axial resolutions of ultra-high-resolution OCT range from 14 μm, with scan widths of 5–12 mm [26,27]. Such a degree of image resolution and definition enables visualization of each layer of the cornea. 

Ultrahigh-resolution OCT machines are the Copernicus HR (Optopol Technologies SA, Zawiercie, Poland) and Bioptigen Envisu (Bioptigen Inc., Morrisville, NC, USA) [22,28]. MS-39 (Costruzione Strumenti Oftalmici, Florence, Italy) is a stand-alone device that combines SD-OCT and Placido disk corneal topography to obtain measurements of the anterior segment of the eye. This device uses a SLED light source at 845 nm and provides an axial resolution of 3.6 μm (in tissue) and transversal resolution of 35 μm (in the air). Each section measures 16 × 7.5 mm and includes 1024 A-scans. Its unique application includes automated measurements of the corneal epithelium, which are accurate and useful in different clinical situations [21,29,30,31].

Swept-source OCT. In 2008, the first SS-OCT was brought to market. The Casia SS-OCT (Tomey, Nagoya, Japan) uses a 1310 nm swept-source laser as a light source, and it was specifically designed to scan the anterior segment with a scan speed of 30,000 A-scans per second, horizontal scan width of 16 mm, axial resolution of 10 μm, and transverse resolution of 30 μm [22]. Triton SS-OCT (Topcon, Tokyo, Japan) is another device that uses a 1050-nm wavelength probe beam and possesses a scan speed of 100,000; A-scans per second and a scan depth of 3 mm, axial resolution of 8 μm, and transverse resolution of 30 μm. Although this OCT is mainly designed for posterior segment investigation, an external add-on lens enables anterior segment imaging. 

## 4. Anterior Segment OCT in Different Corneal Infections

AS-OCT can offer the clinician a quantitative evaluation of several corneal parameters, thanks to its non-contact high-resolution and cross-sectional examination. Considering this, it might be an ideal system for assessing and monitoring the progression of corneal infectious diseases from different pathogens. 

**Figure 4:**
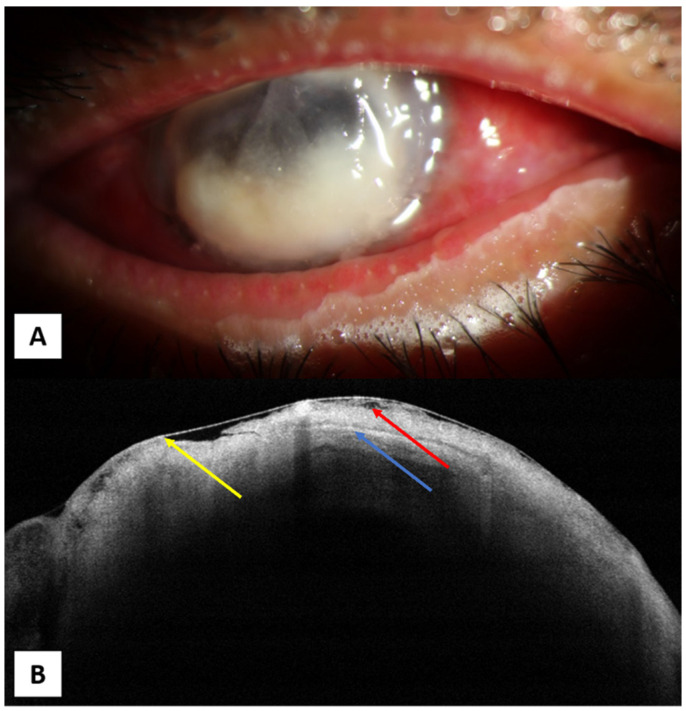
(**A**) A 60-year-old male with fungal keratitis affecting almost the whole cornea. (**B**) AS-OCT showing irregular thickness of the epithelium with areas of epithelial loss (yellow arrow), irregular cystic spaces (red arrow), hyperreflective lesion with different densities affecting the whole corneal thickness (blue arrow).

Bacterial and fungal keratitis. Konstantopoulos et al. measured bacterial keratitis and the results of medical treatments concerning a reduction in corneal inflammation and edema [8,32]. In a study of 26 human bacterial keratitis, both IT and CT significantly decreased after three treatments: these two parameters provided objective evidence of successful medical treatment [8]. In addition to this prompt reduction in IT and CT, resolution of bacterial keratitis was followed by a late reduction in IT. The study authors could measure CT in all patients and IT in 80.8% of patients, but infiltration width could only be calculated in 50% of patients due to two factors: first, infiltrations had soft, poor defined transverse margins, and second, the AS-OCT device used in the study (Visante OCT) had a transverse imaging resolution (60 µm) lower than the axial resolution (18 µm). Infiltration of the corneal stroma appeared as a hyperreflective area in the stroma, making it possible to measure the thickness with caliper tools. Additionally, qualitative evaluation of the infiltration is possible, since the intensity of hyperreflectivity corresponds to the density of the infiltration on slit-lamp examination. Nevertheless, in the later phases of the pathology, the infiltration becomes replaced by scar tissue and measurement of IT may become even more difficult. Corneal edema is imagined as a diffuse thickening of the stroma, with a subsequent change in the convexity of the posterior corneal surface; later on, in more severe cases, Descemet folds can be visualized as ruffles in the usually smooth endothelial surface [32]. Soliman et al. [33], in a prospective observational study of 20 patients with fungal and bacterial microbial keratitis, described a range of characteristic AS-OCT patterns that might be used as an additional instrument in the diagnosis and management of the disease. The ten common morphological patterns described by the authors were hyperreflective lesion in the stroma, epithelial defect, stromal edema, hyperreflective material above the lost epithelium or the hyperreflective stromal lesion, hyperreflective lesion attached to the endothelium surface, localized stromal thinning with an epithelial defect, loss of all layers of the cornea except the Descemet’s membrane, a hyperreflective stromal lesion with intact epithelium with or without stromal thinning (Figure 4), and diffuse stromal thinning with an epithelial defect on top. 

**Figure 5:**
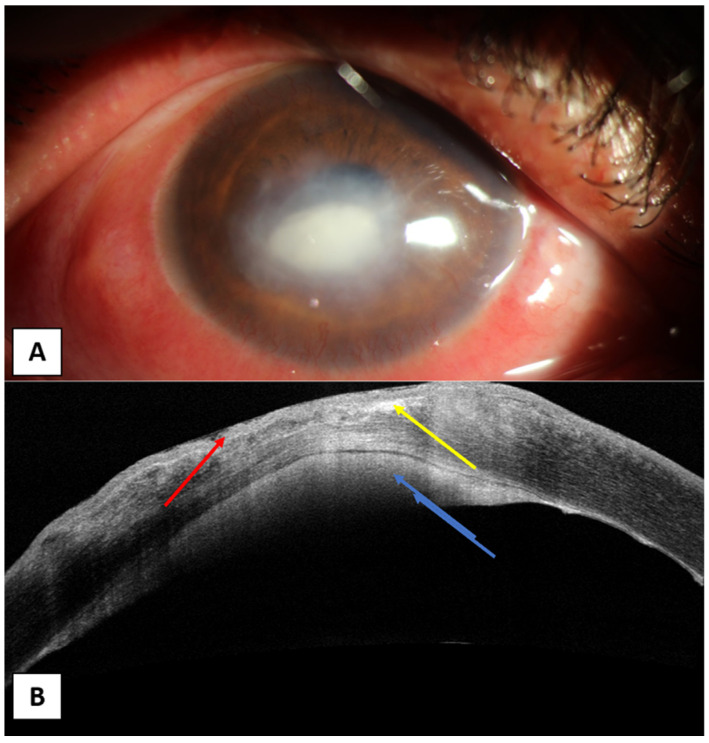
A male patient, 60 years old, presented by history of vegetal trauma to his left eye. (**A**) Slit-lamp photo showing paracentral oval corneal ulcer. (**B**) AS-OCT photo showing irregular epithelium (red arrow) with stromal thinning and irregular hyperreflectivity (yellow arrow). A hyperreflective plaque attached to the back of the cornea is also evident (blue arrow).

Conversely, in cases of fungal keratitis, some unique patterns were present: localized small cystic spaces within the stroma, and full-thickness large cystic spaces within the stroma (evidence of necrotic stroma). Additionally, AS-OCT may help in confirming the exact location of the keratitis, as shown in cases of multiple whitish infiltrates with less defined margins after a lamellar keratoplasty that appeared as high-reflective infiltrates at the graft–host interface at AS-OCT images [34]. AS-OCT might also be useful to demonstrate the exact location and area of the endothelial plaque in patients with fungal keratitis. Indeed, most cases of fungal keratitis showed in AS-OCT an indistinct and irregular boundary among the endothelial plaque and the cornea [35]. This implies that the fungal damage to the endothelium expands from the stroma into the anterior chamber (Figure 5). 

AS-OCT might also be advantageous to guide corneal cross-linking treatment according to the size of the infiltrate and area of inflammation [36].

Acanthamoeba keratitis. Radial keratoneuritis in patients with Acanthamoeba keratitis (AK) may be observed with AS-OCT as highly reflective lines or bands of different widths between 20 and 200 µm, which run into the corneal stroma obliquely or parallel. Such lesions become thinner and eventually disappear after the resolution of the keratitis [37]. Consistent with these findings, Park et al. [38] observed highly reflective bands in the stroma that corresponded to the area of radial keratoneuritis and disappeared as soon as the radial keratoneuritis resolved after proper treatment. However, the authors could not identify Acanthamoeba cysts or trophozoites using AS-OCT because of the limited power resolution of the device. 

Herpes simplex keratitis. In cases of herpes simplex keratitis, corneal infiltrates are imaged on AS-OCT as a lentiform or spindle-shaped hyper-reflective area in the stroma that may be diffuse or localized: images obtained with AS-OCT might help to differentiate between active infiltrates and stromal scarring. Nevertheless, none of the identified AS-OCT features are exclusive of herpetic keratitis, meaning that nowadays AS-OCT can help in the follow-up and management of such pathologies more than in the diagnosis [39]. Hixson et al. reported two cases of disciform keratitis that were followed using AS-OCT to quantify the resolution and decrease of edema throughout the treatment: the thickest area of the cornea was located and measured across time until a complete resolution was achieved. AS-OCT allowed for the objective measurement of therapeutic interventions, resulting in reduced edema and thickness as well as the exact localization of microcystic edema and keratic precipitates [40]. 

Corneal endotheliitis. Coin-shaped lesions are imaged on AS-OCT as an irregularly highly reflective thickened endothelium, as described by Yokogawa et al. [41] in two patients with PCR-proven Cytomegalovirus (CMV) corneal endotheliitis. After anti-CMV treatment, the irregularly thickened highly reflective endothelial cell layer disappeared, indicating that AS-OCT can be useful to image therapeutic effects noninvasively. In a study of 13 eyes with CMV corneal endotheliitis, AS-OCT images showed protruding high-reflective structures at the posterior cornea with a dendritic, dome-shaped, quadrangular, or saw-tooth appearance [42]. In this study, central corneal thickness was followed in seven cases and did not correlate with clinical courses; nevertheless, after proper antiviral treatment, the protruding high-reflective structures disappeared. 

**Figure 6:**
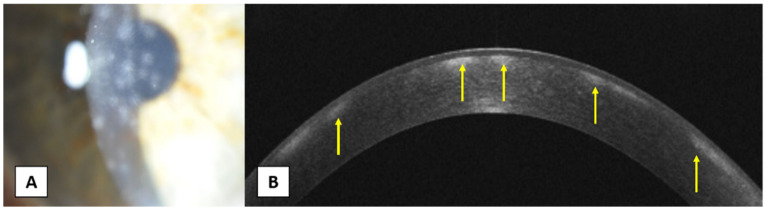
(**A**) A 53-year-old female presents adenoviral nummular infiltrates. (**B**) AS-OCT shows multiple subepithelial lesions with no elevations above the epithelial surface (yellow arrows).

Others. On AS-OCT, the multiple, whitish, raised, epithelial lesions that represent the most common clinical manifestation of ocular microsporidiosis can be seen as raised hyper-reflective epithelial lesions [43]. Thanathanee et al. [44] also reported characteristic images of microsporidia keratoconjunctivitis: AS-OCT images demonstrated hyperreflective dots that were limited to the epithelial layers of the cornea in all cases and were quite raised above the epithelial surface in most of the cases (92.3%). AS-OCT might help in differentiating them from adenovirus nummular scars (hyperreflective lesions started from the subepithelial layer of the cornea and slightly extended into the anterior stroma), which are subepithelial lesions without elevations above the epithelial surface (Figure 6). 

In conclusion, AS-OCT can directly visualize, measure, and monitor in vivo various parameters of corneal infections, especially the depth and extension of corneal thickness, the thickness of the infiltrations, and width. Resolution of corneal infection is characterized by an early reduction in corneal edema, followed by a later reduction in infiltration: both parameters can be routinely measured and followed with AS-OCT, helping in the monitoring of the pathology and the response to the treatment. In clinical practice, serial standardized AS-OCT scanning might be used to quantify and objectively assess the keratitis. Limitations of this diagnostic imaging technology is the impossibility of measuring the density, as this can be a valuable parameter in the follow-up of corneal infections. AS-OCT should be tridimensional in the future to offer more extensive information. Going through details and high-resolution, we can better typify the level of severity and the response to therapy. As far as we know, there are no studies that allow for the quantitative monitoring of these diseases, based on the improvement of the density of the infiltration and infiltration area. OCT shear wave elastography has also emerged as a promising technique with high spatial resolution and high sensitivity to mechanical deformation of tissue, which might give quantitative approaches to corneal infections based on the excitation of elastic waves [45,46]. It has been widely studied with live animals, ex vivo tissues, and healthy subjects, but the ability to measure elastic modulus quantitatively in corneal infections remains to be developed [47,48,49].

It is therefore desirable to develop methods that allow the measurement and monitoring of these two parameters: density of infiltration and infiltration area.

## Data Availability

Data is contained within the article.

## References

[1] Resnikoff S., Pascolini D., Etyaale D., Kocur I., Pararajasegaram R., Pokharel G.P., Mariotti S.P. (2004). Global data on visual impairment in the year 2002. Bull. World Health Organ..

[2] Austin A., Lietman T., Rose-Nussbaumer J. (2017). Update on the Management of Infectious Keratitis. Ophthalmology..

[3] Schaefer F., Bruttin O., Zografos L., Guex-Crosier Y. (2001). Bacterial keratitis: A prospective clinical and microbiological study. Br. J. Ophthalmol..

[4] Hume E.B., Dajcs J.J., Moreau J.M., Sloop G.D., Willcox M.D., O’Callaghan R.J. (2001). Staphylococcus corneal virulence in a new topical model of infection. Investig. Ophthalmol. Vis. Sci..

[5] Bourcier T., Thomas F., Borderie V., Chaumeil C., Laroche L. (2003). Bacterial keratitis: Predisposing factors, clinical and microbiological review of 300 cases. Br. J. Ophthalmol..

[6] Shing Ong H.S., Corbett M.C. (2015). Corneal infections in the 21st century. Postgrad. Med. J..

[7] Efron N., Morgan P.B., Hill E.A., Raynor M.K., Tullo A.B. (2005). The size, location and clinical severity of corneal infiltrative events associated with contact lens wear. Optom. Vis. Sci..

[8] Konstantopoulos A., Kuo J., Anderson D.F., Hossain P.N. (2008). Assessment of the use of anterior segment optical coherence tomography in microbial keratitis. Am. J. Ophthalmol..

[9] Shah A., Sachdev A., Coggon D., Hossain P. (2011). Geographic variations in microbial keratitis: An analysis of the peer-reviewed literature. Br. J. Ophthalmol..

[10] Van der Meulen I.J., van Rooij J., Nieuwendaal C.P., Cleijnenbreugel H.V., Geerards A.J., Remeijer L. (2008). Age-related risk factors, culture outcomes, and prognosis in patients admitted with infectious keratitis to two Dutch tertiary referral centers. Cornea.

[11] Dart J.K., Radford C., Minassian D., Verma S., Stapleton F. (2008). Risk factors for microbial keratitis with contemporary contact lenses. Ophthalmology.

[12] Keay L., Edwards K., Naduvilath T., Taylor H.R., Snibson G.R., Forde K., Stapleton F. (2006). Microbial keratitis predisposing factors and morbidity. Ophthalmology.

[13] Loh R.S., Chan C.M., Ti S.E., Lim L., Chan K.S., Tan D.T. (2009). Emerging prevalence of microsporidial keratitis in Singapore: Epidemiology, clinical features, and management. Ophthalmology.

[14] Das S., Sharma S., Sahu S.K., Nayak S.S., Kar S. (2012). Diagnosis, clinical features and treatment outcome of microsporidial keratoconjunctivitis. Br. J. Ophthalmol..

[15] Dart J.K., Saw V.P., Kilvington S. (2009). Acanthamoeba keratitis: Diagnosis and treatment update 2009. Am. J. Ophthalmol..

[16] Gellrich M.M., Gellrich M.-M. (2014). The slit lamp: Applications for biomicroscopy and videography. History of the Slit Lamp.

[17] Allan D.S., Dart J.K.G. (1995). Strategies for the management of microbial keratitis. Br. J. Ophthalmol..

[18] Eleinen K.G., Mohalhal A.A., Elmekawy H.E., Abdulbaki A.M., Sherif A.M., El-Sherif R.H., Rahman E.M.A. (2012). Polymerase chain reaction-guided diagnosis of infective keratitis—A hospital-based study. Curr. Eye Res..

[19] Wang S.B., Cornish E.E., Grigg J.R., McCluskey P.J. (2019). Anterior segment optical coherence tomography and its clinical applications. Clin. Exp. Optom..

[20] Ang M., Baskaran M., Werkmeister René M., Chua J., Schmidl D., Aranha dos Santos V., Garhöfer G., Mehta J.S., Schmetterer L. (2018). Anterior segment optical coherence tomography. Prog. Retin. Eye Res..

[21] Alio J.L., del Barrio J.L.A. (2021). Atlas of Anterior Segment Optical Coherence Tomography. Essentials in Ophthalmology.

[22] Han S.B., Liu Y.C., Noriega K.M., Mehta J.S. (2016). Applications of anterior segment optical coherence tomography in cornea and ocular surface diseases. J. Ophthalmol..

[23] Shan J., DeBoer C., Xu B.Y. (2019). Anterior Segment Optical Coherence Tomography: Applications for Clinical Care and Scientific Research. Asia Pac. J. Ophthalmol..

[24] Ramos J.L.B., Li Y., Huang D. (2009). Clinical and research applications of anterior segment optical coherence tomography—A review. Clin. Exp. Ophthalmol..

[25] Bald M., Li Y., Huang D. (2012). Anterior chamber angle evaluation with fourier-domain optical coherence tomography. J. Ophthalmol..

[26] Wang J., Abou Shousha M., Perez V.L., Karp C.L., Yoo S.H., Shen M., Cui L., Hurmeric V., Du C., Zhu D. (2011). Ultra-high resolution optical coherence tomography for imaging the anterior segment of the eye. Ophthalmic Surg Lasers Imaging.

[27] Shousha M.A., Perez V.L., Wang J., Ide T., Jiao S., Chen Q., Chang V., Buchser N., Dubovy S.R., Feuer W. (2010). Use of ultrahigh- resolution optical coherence tomography to detect in vivo characteristics of Descemet’s membrane in Fuchs’ dystrophy. Ophthalmology.

[28] Thomas B.J., Galor A., Nanji A.A., Sayyad F.E., Wang J., Dubovy S.R., Joag M.G., Karp C.L. (2014). Ultra highresolution anterior segment optical coherence tomography in the diagnosis and management of ocular surface squamous neoplasia. Ocul. Surf..

[29] Abdelghany A., D’Oria F., Alio J.L. (2021). Surgery of glaucoma in modern corneal graft procedures. Surv. Ophthalm..

[30] Del Barrio J.L.A., D’Oria F., Alio J.L. (2021). Visian Implantable Collamer Lens Behavior in Descemet’s Membrane Endothelial Keratoplasty Surgery. Cornea.

[31] D’Oria F., Alio J.L., Rodriguez A.E., Amesty M.A., Abu-Mustafa S.K. (2021). Cosmetic Keratopigmentation in Sighted Eyes: Medium and Long-Term Clinical Evaluation. Cornea.

[32] Konstantopoulos A., Yadegarfar G., Fievez M., Anderson D.F., Hossain P. (2011). In vivo quantification of bacterial keratitis with optical coherence tomography. Investig. Ophthalmol. Vis. Sci..

[33] Soliman W., Fathalla A.M., El-Sebaity D.M., Al-Hussaini A.K. (2013). Spectral domain anterior segment optical coherence tomography in microbial keratitis. Graefes. Arch. Clin. Exp. Ophthalmol..

[34] D’Oria F., Galeone A., Pastore V., Cardascia N., Alessio G. (2019). Multi-drug resistant Enterococcus faecium in late-onset keratitis after deep anterior lamellar keratoplasty: A case report and review of the literature. Medicine.

[35] Takezawa Y., Suzuki T., Shiraishi A. (2017). Observation of Retrocorneal Plaques in Patients with Infectious Keratitis Using Anterior Segment Optical Coherence Tomography. Cornea.

[36] Abbouda A., Estrada A.V., Rodriguez A.E., Alio J.L. (2014). Anterior segment optical coherence tomography in evaluation of severe fungal keratitis infections treated by corneal crosslinking. Eur. J. Ophthalmol..

[37] Yamazaki N., Kobayashi A., Yokogawa H., Ishibashi Y., Oikawa Y., Tokoro M., Sugiyama K. (2014). In vivo imaging of radial keratoneuritis in patients with Acanthamoeba keratitis by anterior-segment optical coherence tomography. Ophthalmology.

[38] Park Y.M., Lee J.S., Yoo J.M., Park J.M., Seo S.W., Chung I.Y., Kim S.J. (2018). Comparison of anterior segment optical coherence tomography findings in acanthamoeba keratitis and herpetic epithelial keratitis. Int. J. Ophthalmol..

[39] Soliman W., Nassr M.A., Abdelazeem K., Al-Hussaini A.K. (2019). Appearance of herpes simplex keratitis on anterior segment optical coherence tomography. Int. Ophthalmol..

[40] Hixson A., Blanc S., Sowka J. (2014). Monitoring keratitis resolution with optical coherence tomography. Optom. Vis. Sci..

[41] Yokogawa H., Kobayashi A., Yamazaki N., Sugiyama K. (2014). In vivo imaging of coin-shaped lesions in cytomegalovirus corneal endotheliitis by anterior segment optical coherence tomography. Cornea.

[42] Kobayashi R., Hashida N., Soma T., Koh S., Miki A., Usui S., Maeda N., Nishida K. (2017). Clinical Findings of Anterior Segment Spectral Domain Optical Coherence Tomography Images in Cytomegalovirus Corneal Endotheliitis. Cornea.

[43] Sridhar M.S., Shaik B. (2018). Anterior segment optical coherence tomography of microsporidial keratoconjunctivitis. Indian J. Ophthalmol..

[44] Thanathanee O., Laohapitakvorn S., Anutarapongpan O., Suwan-Apichon O., Bhoomibunchoo C. (2019). Anterior Segment Optical Coherence Tomography Images in Microsporidial Keratoconjunctivitis. Cornea.

[45] Ramier A., Eltony A.M., Chen Y., Clouser F., Birkenfeld J.S., Watts A., Yun S.H. (2020). In vivo measurement of shear modulus of the human cornea using optical coherence elastography. Sci. Rep..

[46] Ford M.R., Dupps W.J., Rollins A.M., Roy A.S., Hu Z. (2011). Method for optical coherence elastography of the cornea. J. Biomed. Opt..

[47] De Stefano V.S., Ford M.R., Seven I., Dupps W.J. (2018). Live human assessment of depth-dependent corneal displacements with swept-source optical coherence elastography. PLoS ONE.

[48] Han Z., Li J., Singh M., Wu C., Liu C.-H., Raghunathan R., Aglyamov S.R., Vantipalli S., Twa M., Larin K.V. (2017). Optical coherence elastography assessment of corneal viscoelasticity with a modified Rayleigh-Lamb wave model. J. Mech. Behav. Biomed. Mater.

[49] Li J., Singh M., Aglyamov S., Emelianov S., Twa M.D., Larin K.V. (2014). Air-pulse OCE for assessment of age-related changes in mouse cornea in vivo. Laser Phys. Lett..

